# Supplementary figures and images for: Circulating microRNA/isomiRs as novel biomarkers of esophageal squamous cell carcinoma
Source: PLoS One. 2020 Apr 6;15(4):e0231116. doi: 10.1371/journal.pone.0231116 (PMC7135252; doi:10.1371/journal.pone.0231116)

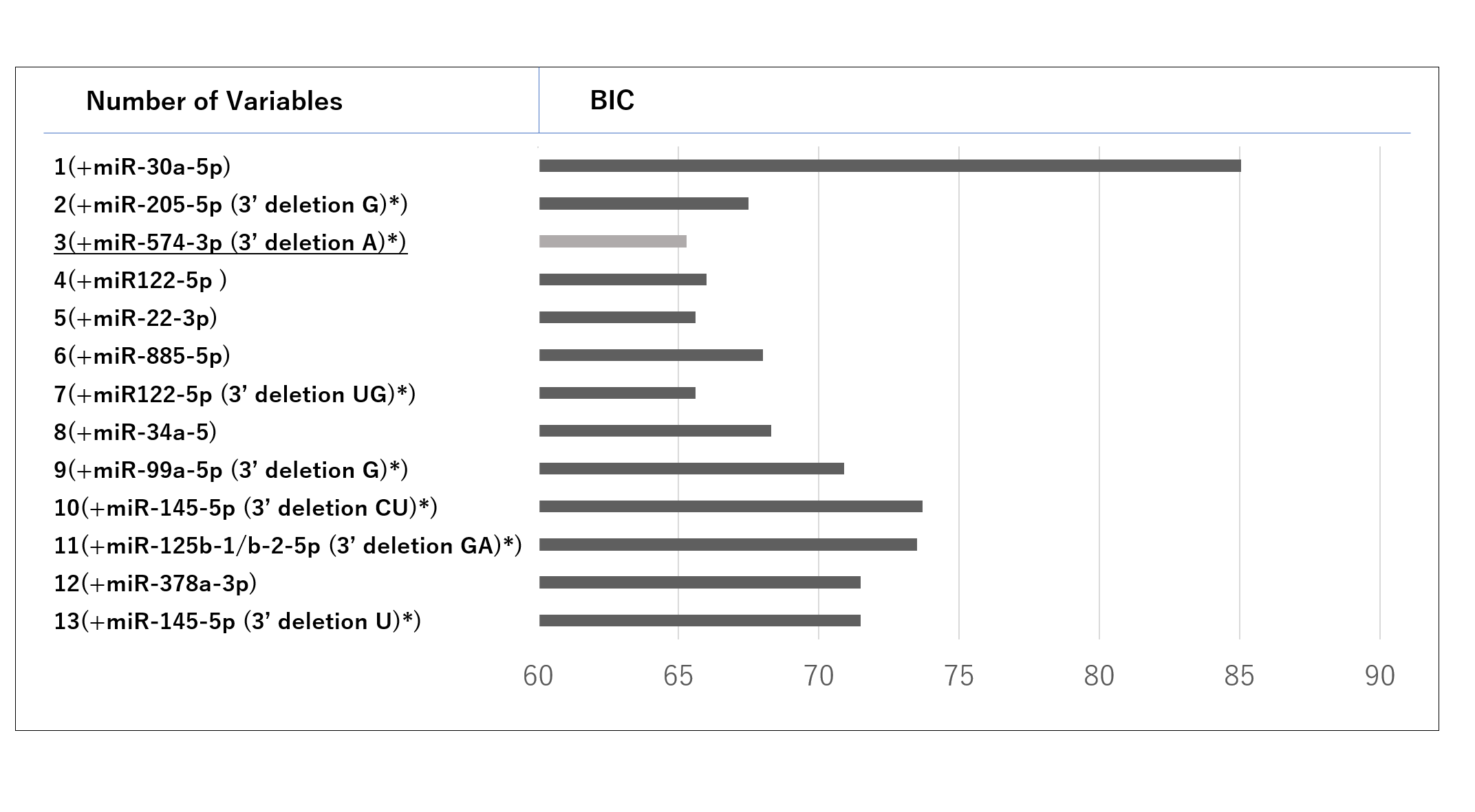

Supplement: S1 Fig — The forward stepwise model showed the combination of miR-574-3p (3’ deletion A), miR-205-5p (3’ deletion G), and miR-30a-5p indicated the minimum BIC. (TIF) [file pone.0231116.s001.tif]

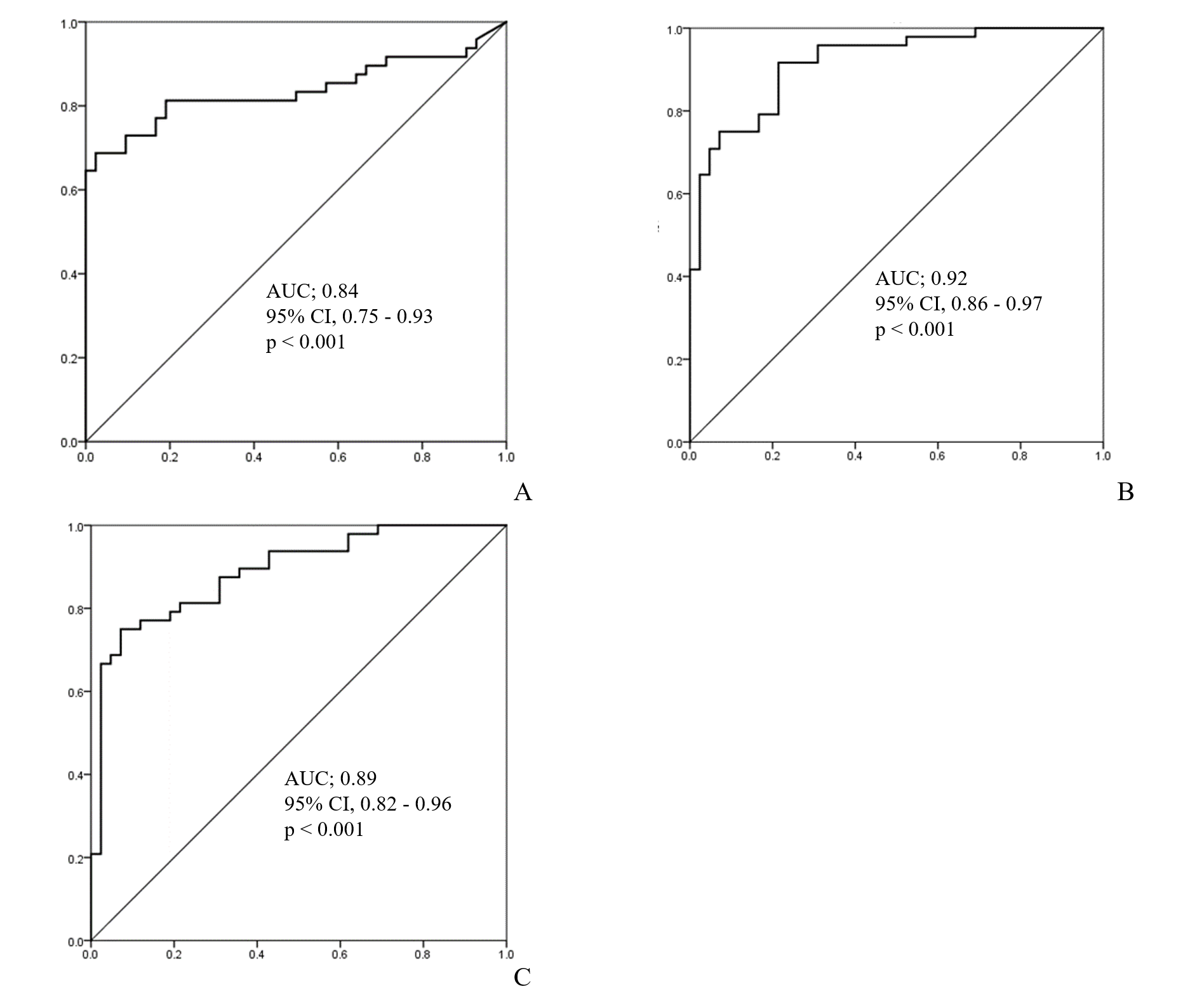

Supplement: S2 Fig — Area under the receiver operating characteristics curves (AUC) for miR-574-3p (3’ deletion A) (A), miR-205-5p (3’ deletion G) (B), and miR-30a-5p (C) to predict esophageal squamous cell carcinoma. miR-574-3p (3’ deletion A): AUC, 0.84; 95% CI, 0.75–0.93; p<0.001; miR-205-5p (3’ deletion G): AUC, 0.92; 95% CI, 0.86–0.97; p<0.001, and miR-30a-5p: AUC, 0.89; 95% CI, 0.82–0.96; p<0.001. (TIF) [file pone.0231116.s002.tif]

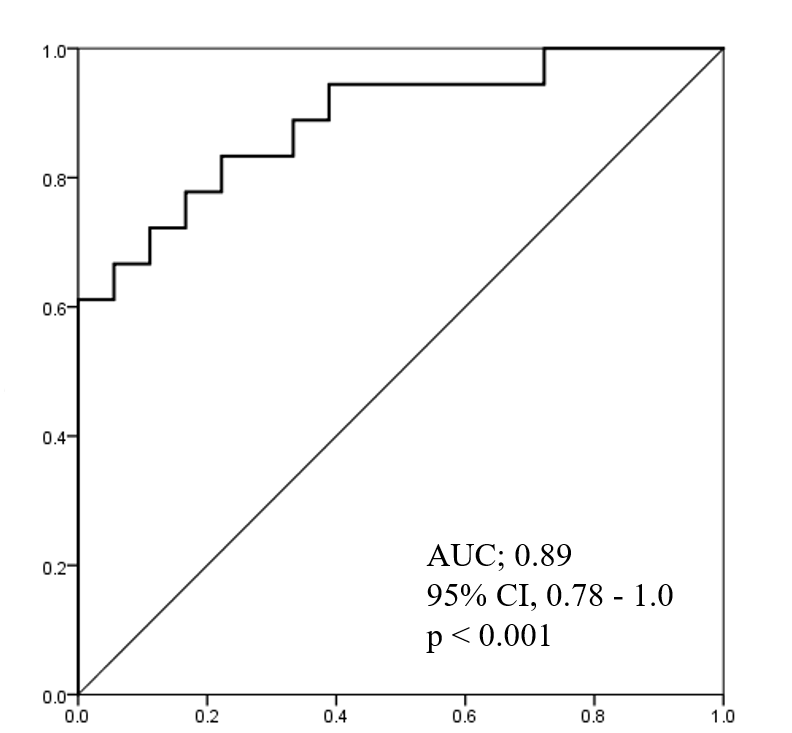

Supplement: S3 Fig — Area under the receiver operating characteristics curves (AUC) for the panel index to predict esophageal squamous cell carcinoma: AUC, 0.89; 95% CI, 0.78–1.0; p<0.001. (TIF) [file pone.0231116.s003.tif]

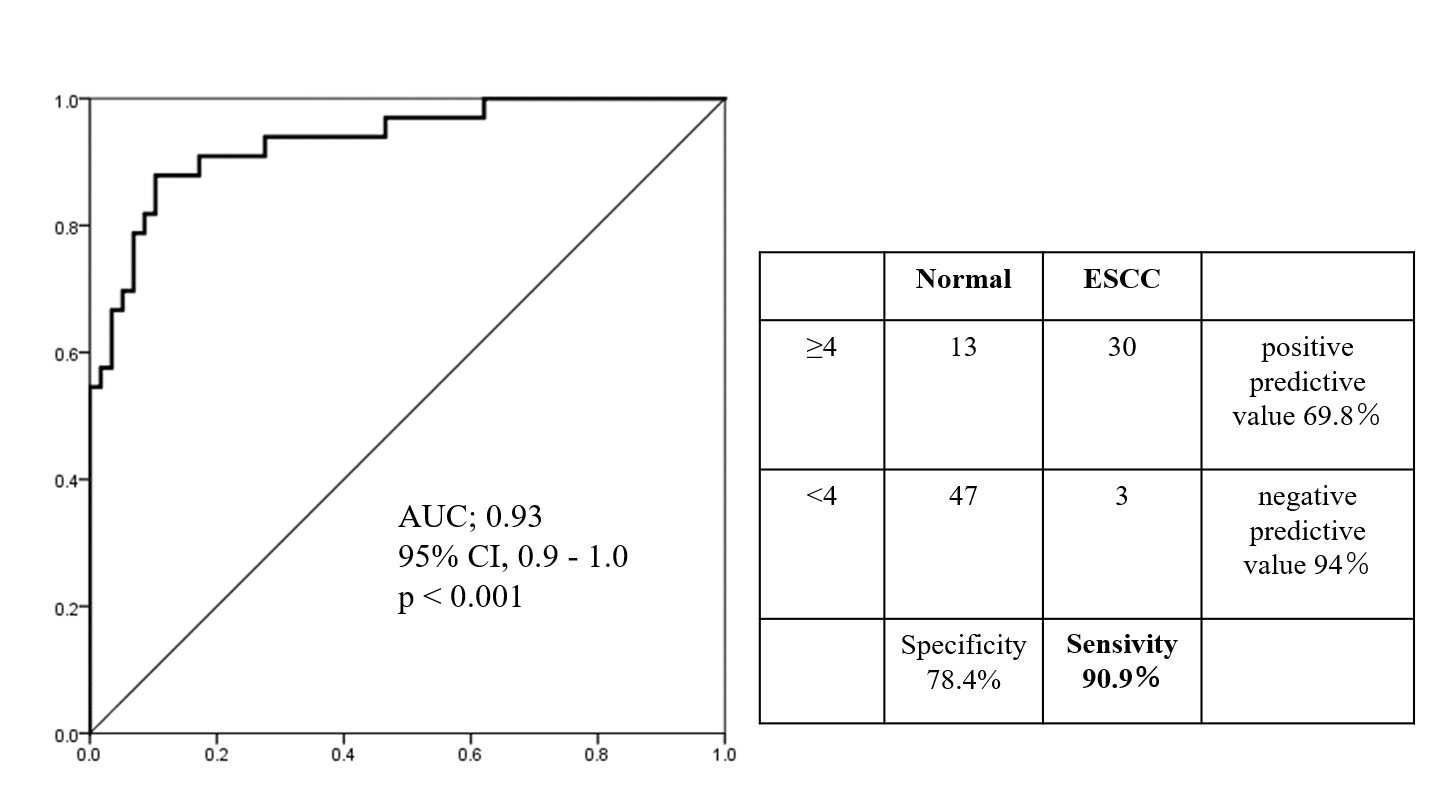

Supplement: S4 Fig — Area under receiver operating characteristics curves (AUC) for panel index to predict stage I esophageal squamous cell carcinoma: AUC, 0.93; 95% CI, 0.9–1.0; p<0.001. Diagnostic sensitivity and specificity were 90.4% and 78.4%, using a cut off value for the panel index of 4.0. (TIF) [file pone.0231116.s004.tif]

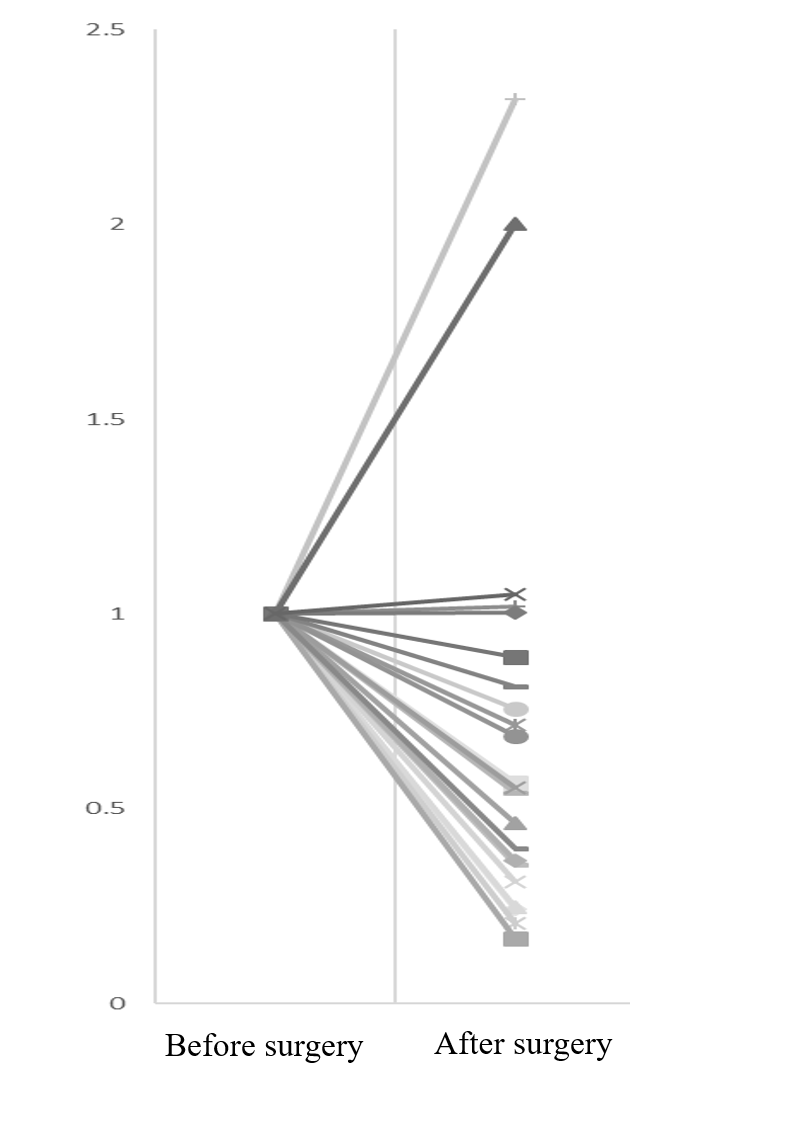

Supplement: S5 Fig — Change rate of the panel index when the pre-treatment panel index is 1.0. Mean post-treatment panel index was significantly decreased compared with pre-treatment (mean decrease in ratio was 0.28±0.15). (TIF) [file pone.0231116.s005.tif]

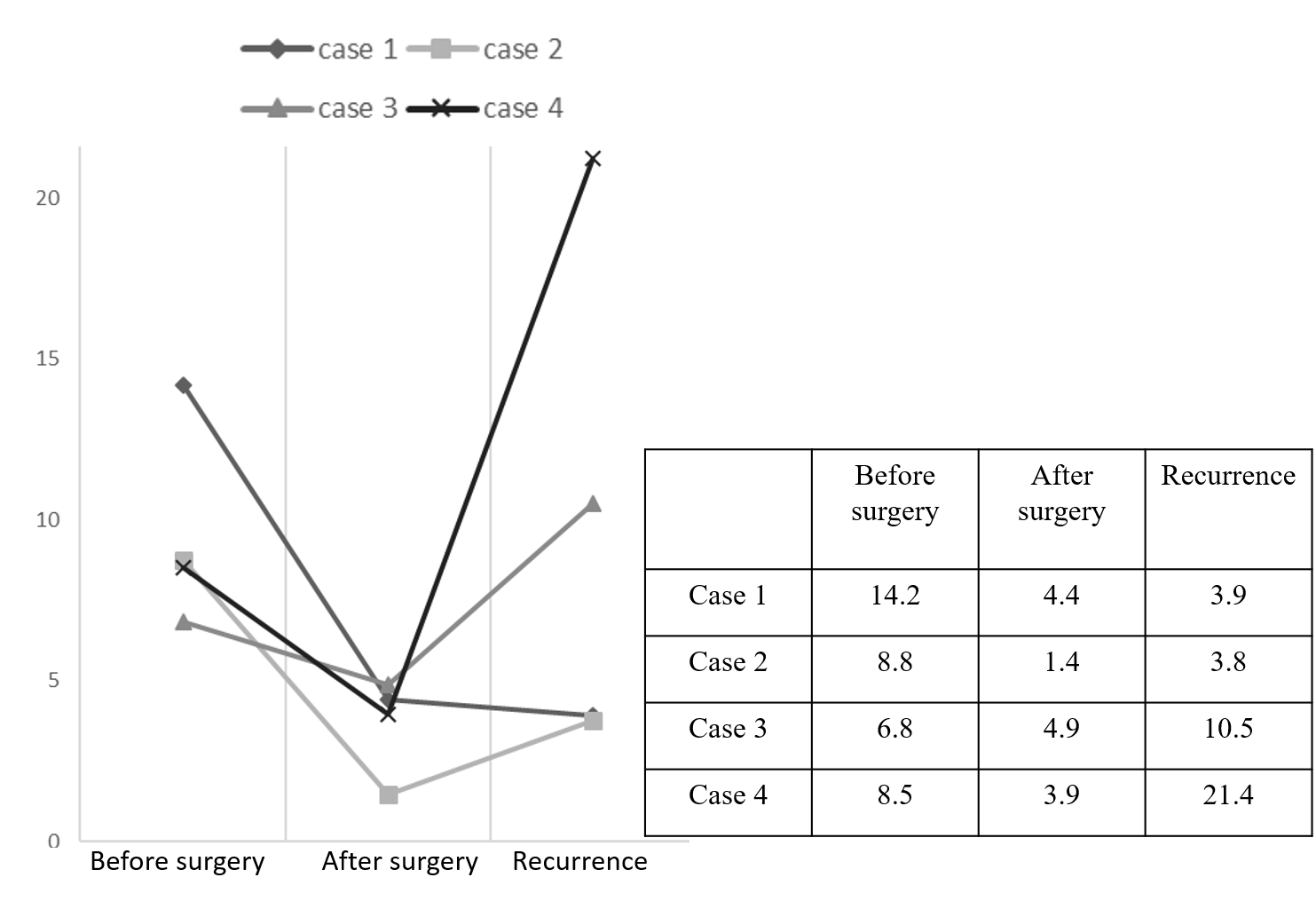

Supplement: S6 Fig — Time course of changes in panel index in patients who experienced post-operative recurrence. (TIF) [file pone.0231116.s006.tif]
